# Collaborative Quality Improvement in the Congenital Heart Defects: Development of the ASSIST Consortium and a Preliminary Surgical Outcomes Report

**DOI:** 10.21470/1678-9741-2016-0074

**Published:** 2017

**Authors:** Fabio Carmona, Paulo Henrique Manso, Mariana Nicoletti Ferreira, Nana Miura Ikari, Marcelo Biscegli Jatene, Luciana Amato, Aida Luiza Turquetto, Luiz Fernando Caneo

**Affiliations:** 1 Hospital das Clinicas of Faculdade de Medicina de Ribeirão Preto of Universidade de São Paulo (FMRP-USP), Ribeirão Preto, SP, Brazil; 2 Heart Institute of Hospital das Clínicas of Faculdade de Medicina of Universidade de São Paulo (InCor-HCFMUSP), São Paulo, SP, Brazil

**Keywords:** Heart Defects, Congenital, Quality Improvement, Database, Cardiovascular Surgical Procedures

## Abstract

**Objective:**

ASSIST is the first Brazilian initiative in building a collaborative quality improvement program in pediatric cardiology and congenital heart disease. The purposes of this manuscript are:

(a) to describe the development of the ASSIST project, including the historical, philosophical, organizational, and infrastructural components that will facilitate collaborative quality improvement in congenital heart disease care;
(b) to report past and ongoing challenges faced; and
(c) to report the first preliminary data analysis.

**Methods:**

A total of 614 operations were prospectively included in a comprehensive online database between September 2014 and December 2015 in two participating centers. Risk Adjustment for Congenital Heart Surgery (RACHS) 1 and Aristotle Basic Complexity (ABC) scores were obtained. Descriptive statistics were provided, and the predictive values of the two scores for mortality were calculated by multivariate logistic regression models.

**Results:**

Many barriers and challenges were faced and overcome. Overall mortality was 13.4%. Independent predictors of in-hospital death were: RACHS-1 categories (3, 4, and 5/6), ABC level 4, and age group (≤ 30 days, and 30 days - 1 year).

**Conclusion:**

The ASSIST project was successfully created over a solid base of collaborative work. The main challenges faced, and overcome, were lack of institutional support, funding, computational infrastructure, dedicated staff, and trust. RACHS-1 and ABC scores performed well in our case mix. Our preliminary outcome analysis shows opportunities for improvement.

**Table:** 

Abbreviations, acronyms & symbols				
ABC	= Aristotle Basic Complexity		LR	= Likelihood ratio
ACC	= Aristotle Comprehensive Complexity		PC4	= Pediatric Cardiac Critical Care Consortium
AUC	= Area under the curve		RACHS	= Risk Adjustment for Congenital Heart Surgery
CHD	= Congenital heart disease		REDCap	= Research Electronic Data Capture
CHSS	= Congenital Heart Surgeons Society		ROC	= Receiver-operator characteristics
CNPq	= National Council for Scientific and Technological Development		SIR	= Standardized infection ratio
DATASUS	= National Health System database		SMR	= Standardized mortality ratios
FAPESP	= São Paulo State Foundation for Research Support		STS	= Society of Thoracic Surgeons
IaaS	= Infrastructure as a service		USP	= University of São Paulo
IQIC	= International Quality Improvement Collaborative		VIS	= Vasoactive-inotropic scores

## INTRODUCTION

Treatment of congenital heart disease has evolved in the last decades, with many technical and technological advances. However, significant variation in main outcomes is still present, especially in developing countries. In addition, large countries such as Brazil, a country of continental dimensions, may have many significant regional differences. Establishing objective parameters to evaluate quantitative and qualitative results, and benchmarking them against those of developed countries, is a big challenge.

Collaborative quality improvement programs have contributed to improving the quality of healthcare in many different scenarios. This is because they help to target reasons for such variations and to find solutions for shared problems. In fact, joining a collaborative quality improvement program has proved beneficial in different countries^[1-3]^. Other advantages of joining such programs are improvements in long-term survival and better use of resources. There is a great number of examples of successful programs, such as the Pediatric Cardiac Critical Care Consortium (PC4), which includes some of the best USA pediatric cardiac centers, and the International Quality Improvement Collaborative (IQIC), designed as a consortium focused on developing countries doing pediatric cardiac surgery. They have in common a strong database, a powerful data analysis center, and a structure based on the Learning Health System model described by the Institute of Medicine, in which patients, clinicians, researchers, and other stakeholders collaborate in a meaningful partnership to improve outcomes and generate new knowledge, and where healthcare improvement and research are purposefully integrated^[4,5]^.

ASSIST is the first Brazilian initiative in building a collaborative quality improvement program in pediatric cardiology. The purposes of this manuscript are:

(a) to describe the development of ASSIST, including the historical, philosophical, organizational, and infrastructural components that will facilitate collaborative quality improvement in congenital heart disease (CHD) care;
(b) to report past and ongoing challenges faced; and
(c) to report the first preliminary data analysis.

### Development of the ASSIST Registry

The idea of a Brazilian consortium on outcomes of heart surgeries for CHD was born in 2013 during discussions between researchers from two hospitals linked to the University of São Paulo (USP). It was clear that they were looking for solutions for common problems and that they could learn from each other as well as from combined analyses of their data. The first step was then to build a multi-site network, practice-based registry data and a web-based data center to report participant outcomes, establishing a benchmark to be followed by their centers. ASSIST collaborated to form a consortium focused on standardizing data collection for the care of CHD patients across institutions and defining quality metrics for clinical practice. The consortium was initially funded by the Brazilian Ministry of Health, the National Council for Scientific and Technological Development (CNPq), and the São Paulo State, all through the São Paulo State Foundation for Research Support (FAPESP).

The philosophy behind ASSIST is that sharing knowledge, expertise, and clever solutions for common problems can lead to better outcomes in pediatric heart surgery for CHD. ASSIST is organized as follows: a main core of specialists, including clinicians, surgeons, and respiratory therapists; a statistics team; and partners. Each partner institution has three leaders: an administrator, a physician, and a nurse. There are many other members who are responsible for data collection and audit. Infrastructure is completely online.

## METHODS

The study was approved by the Institutional Review Board of the two participating hospitals, with a waiver from obtaining informed consent. A series of in-person and online meetings took place to standardize data collection, discuss barriers and challenges, and propose solutions for them. After careful planning and documentation, prospective data collection started in September 2014 in two tertiary-care university hospitals. The instruments for data collection were designed by the researchers and included a comprehensive set of pre-, intra- and postoperative variables. This report included data from then up to December 2015. At the time data was collected, some patients were still hospitalized and were therefore not included here. Vasoactive-inotropic scores (VIS) were calculated according to Gaies et al.^[6]^. Procedural complexity was categorized using Risk Adjustment for Congenital Heart Surgery (RACHS)-1 categories and Aristotle Basic Complexity (ABC) scores and levels, whenever possible^[7,8]^. Those two methods have been extensively used in the specialized literature to estimate procedure-specific complexity.

### Statistical Analysis

Results are expressed as mean ± standard deviation, median (total range), or count (proportion), as appropriate. Mortality was calculated based on surgeries: if a patient was submitted to another surgery within the same hospital admission, then a competing outcome (reoperation) was assumed for the first surgery. Single comparisons between hospitals were done using Student's t test, Mann-Whitney's U test, of Fisher's exact test, as appropriate.

The predictive value of the two scores, RACHS-1 and ABC, for in-hospital mortality was assessed using receiver-operator characteristics (ROC) curves and their area under the curve (AUC, a.k.a. the c statistics).

Mortality was also modeled using multivariate logistic regression models with in-hospital mortality as the dependent variable and RACHS-1 categories or ABC levels as independent variables, with age group (≤ 30 days, < 1 year, < 18 years), major noncardiac structural anomaly, prematurity, and combination procedure as covariates. Only patients with an assigned RACHS-1 category or ABC level and age at surgery < 18 years were included in the modeling. Significance and goodness-of-fit were assessed by likelihood ratio (LR) test, chi-square (Hosmer-Lemeshow) goodness-of-fit, and analysis of residuals. Effect size was measured by Nagelkerke's R^2^. Reliability was assessed by expected dispersion (actual *vs.* expected variances) and predicted *versus* observed correlation coefficient. Predictive ability was assessed by the AUC the ROC curve.

The sum of the probability of death of all individuals, as calculated from the model with RACHS-1 categories, resulted in the expected number of deaths. Standardized mortality ratios (SMR) were calculated for the two hospitals by dividing the observed mortality rate by the expected mortality rate.

All analyses were done in IBM SPSS 20.0 (Chicago, IL, USA). *P*-values of 0.05 or less were considered statistically significant.

## RESULTS

### Barriers, Challenges and Solutions

While planning this initiative, we faced many challenges, both anticipated and non-anticipated. Here we describe how some of those challenges were faced, the solutions found, and which ones are still being faced.

### Lack of Institutional Support

Before we started this project, a strong culture of data-driven quality improvement was present only in one of the hospitals. Nevertheless, this culture relied on individual efforts rather than an institutional commitment. During the planning of this project, administrators of both institutions were contacted and assured institutional support. A culture of quality improvement, however, would have to be created and sustained.

### Lack of Funding

We applied for a grant from the Brazilian Ministry of Health, the CNPq, and the São Paulo State, all through the FAPESP. The grant was used mainly to support data collection and auditing.

### Lack of Computational Infrastructure

A robust computational infrastructure was needed. Setting up our own infrastructure would be costly and would require dedicated information technology staff. This problem was solved by hosting the database in a virtual server (infrastructure as a service [IaaS]) provided by the USP (internuvem.usp.br). InterNuvem provides storage and high-performance computational services for researchers linked to the USP. We also needed a software platform in which to build the database. Among the available options, we decided to use Research Electronic Data Capture (REDCap)^[9]^, which is a secure web application for building and managing online surveys and databases. It is specifically geared to support online or offline data capture for research studies and operations. This platform performed very well, with elevated uptime, flexibility and scalability.

### Lack of Dedicated Staff to Data Collection

By the time the project was started, data collection was being done by the researchers themselves. In Brazil, the number of healthcare staff is limited, which precludes allocation of professionals exclusively to data collection. After the grant was obtained, we hired temporary, dedicated personnel for data collection.

### Lack of Trust between Different Teams and Hospitals

Reporting data is not part of our culture and measuring outcomes is not a common practice in our country. Currently, it is very difficult to evaluate and compare performances of different centers because of various reasons, including: lack of a national database, lack of structured forms for collecting data, lack of a common nomenclature for heart defects and procedures, among others. In this scenario, a great number of practitioners, physicians, and the whole team do not feel comfortable having their results publicly reported. With ASSIST, we tried to address many of these issues.

### Preliminary Analysis: Case Mix

**Fig. 1:**
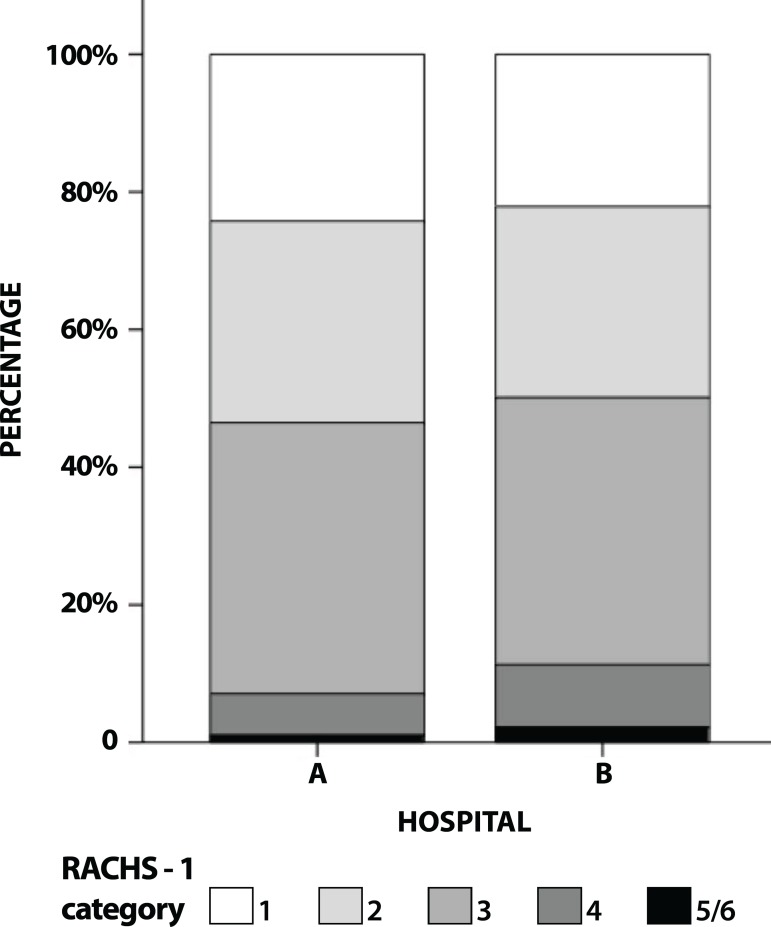
Distribution of cases of the two hospitals according to the Risk Adjustment for Congenital Heart Surgery (RACHS) 1 categories.

A total of 614 operations were collected in this period. Demographic and preoperative clinical data are shown in Table 1. Distribution of diagnosis is shown in Table 2. Distribution of cases according to RACHS-1 categories is shown in Figure 1.

**Table 1:** Preoperative demographic and clinical data.

Variable	Hospital A (N=106)	Hospital B (N=508)
Age at surgery	6 m (4 d - 16 y)	17 m (0 d - 66 y)
Gender (male)	60 (56%)	271 (53%)
Weight (kg)	5.15 (0.65-59.55)	9.20 (1.00-102.00)
Weight-for-age Z-score < -2	40 (37.7%)	161 (37.1%)
Length or height (cm)	62 (32-159)	79 (36-183)
BMI (kg/m^2^)	13.9 (6.5-24.3)	15.6 (6.8-37.5)
BMI-for-age Z-score < -2	29 (27.4%)	101 (23.3%)
Prenatal diagnosis	8 (9.2%)	64 (16.6%)
Number of previous surgeries	0 (0-5)	0 (0-5)
Prematurity	21 (19.8%)	32 (6.3%)
Major noncardiac structural anomaly	0	3 (0.6%)
Combination procedure	12 (11.3%)	28 (5.5%)
Preoperative hematocrit (%)	35 (23-63)	40 (12-65)
Preoperative SaO_2_ (%)	93 (45-100)	96 (29-100)

BMI=body mass index; SaO_2_=arterial oxygen saturation; m=months; d=days; y=years Values are expressed as median (range) or count (proportion).

**Table 2:** Frequency of diagnoses by group.

Diagnosis	Frequency	Percentage
Single ventricle	94	15.3
Tetralogy of Fallot	82	13.4
ASD	81	13.2
VSD	71	11.6
AV canal	50	8.1
Pulmonary atresia	43	7.0
Coarctation of aorta and aortic arch hypoplasia	33	5.4
Patent ductus arteriosus	25	4.1
Transposition of the great arteries	25	4.1
DORV	19	3.1
Partial anomalous pulmonary venous connection	16	2.6
Aortic valve disease	14	2.3
Mitral valve disease	9	1.5
Cardiomyopathy	8	1.3
Pulmonary valve disease	8	1.3
Truncus arteriosus	7	1.1
Tricuspid valve disease and Ebstein's anomaly	5	0.8
Hypoplastic left heart syndrome	4	0.7
RVOT obstruction and/or pulmonary stenosis	4	0.7
Total anomalous pulmonary venous connection	3	0.5
Unassigned	2	0.3
Shone's syndrome	2	0.3
Aortic aneurysm	1	0.2
AP window	1	0.2
Conduit failure	1	0.2
Congenitally corrected TGA	1	0.2
Cor triatriatum	1	0.2
Electrophysiological	1	0.2
LV to aorta tunnel	1	0.2
Miscellaneous, other	1	0.2
Vascular rings and Slings	1	0.2

ASD=atrial septal defect; VSD=ventricular septal defect; AV=atrioventricular; DORV=double outlet left ventricle; RVOT=right ventricle outflow tract; AP=aortopulmonary; TGA=transposition of great arteries; LV=left ventricle

Patients were referred from different states within the country, mostly from the state of São Paulo. A map of the cities of origin of all patients can be found at https://www.mapcustomizer.com/map/ASSIST.

### Outcome Analysis: Mortality

**Fig. 2:**
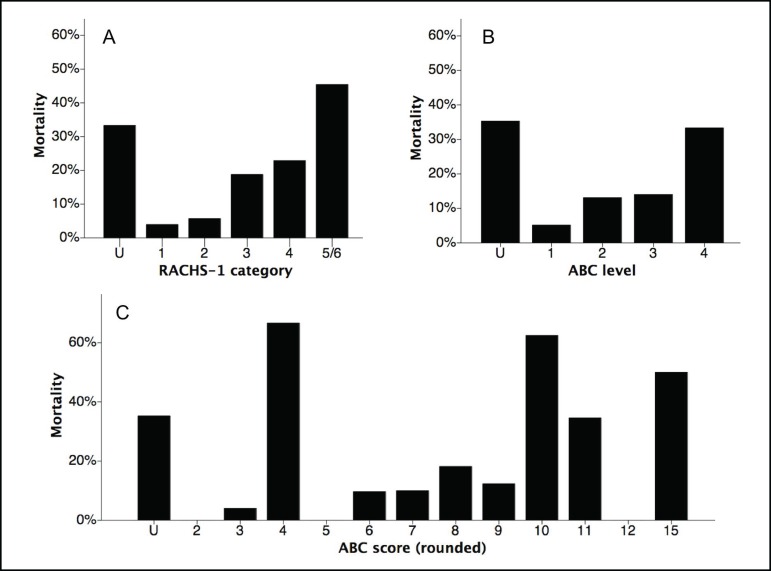
Mortality according to Risk Adjustment for Congenital Heart Surgery (RACHS) 1 categories (A), and Aristotle Basic Complexity (ABC) levels (B) and rounded scores (C). U=undefined

Surgical and postoperative data are shown in Table 3. Overall mortality was 13.4%. Mortality was 17.1% in hospital A and 12.5% in hospital B (*P*=NS), for a predicted mortality of 14.6% and 13.1%, respectively. Figure 2 shows mortality according to RACHS-1 categories and ABC levels and rounded scores. The AUC at ROC curves of RACHS-1 and ABC score were 0.699 and 0.650, respectively, on predicting mortality.

**Table 3:** Surgical and postoperative data.

Variable	Hospital A (N=106)	Hospital B (N=508)
CPB duration (min)	100 (0-390)	84 (0-365)
XAo duration (min)	55 (0-172)	45 (0-325)
DHCA duration (min)	0 (0-145)	0 (0-150)
Intraoperative death	1 (0.9%)	7 (1.4%)
Complications		
Bleeding	12 (11.7%)	37 (7.3%)
Arrhythmia	4 (3.8%)	23 (4.5%)
Postoperative hematocrit (%)	33 (10-59)	37 (15-61)
Postoperative arterial lactate (mg/dL)	18 (1.8-243)	24 (6-270)
VIS at the end of surgery	5 (0-55)	10 (0-325)
VIS at CICU arrival	8 (0-54)	32 (3-70)
Length of mechanical ventilation (h)	90 (0-1055)	17 (0-8779)
Length of CICU stay (h)	141 (2-1885)	136 (16-8879)

CPB=cardiopulmonary bypass; XAo=aortic cross-clamp; DHCA=deep hypothermic circulatory arrest; VIS=vasoactive-inotropic score; CICU=cardiac intensive care unit Values are expressed as median (range) or count (proportion).

### Modeling in-Hospital Mortality: Independent Effects of RACHS-1 and ABC

A total of 491 surgeries could be assigned to a RACHS-1 category. These were included in the model, which is shown in Table 4. In this model, the independent predictors of in-hospital death were RACHS-1 categories (3, 4, and 5/6) and age (≤ 30 days and 30 days - 1 year).

**Table 4:** Multivariate logistic regression of in-hospital mortality using RACHS-1 categories (n=491).

	OR	95%CI		*P*-value
		Lower	Upper	
RACHS-1 category				0.001
1	1.000			-
2	0.998	0.297	3.351	0.997
3	4.050	1.444	11.353	0.008
4	2.844	0.825	9.801	0.098
5/6	10.970	2.173	55.378	0.004
Age group				0.000
≤ 30 days	13.132	5.357	32.187	0.000
30 days - 1 year	3.678	1.720	7.867	0.001
1 - 18 years				-
Prematurity	0.865	0.331	2.260	0.767
Major noncardiac structural anomaly	6.962	0.094	515.096	0.377
Combination procedure	1.325	0.490	3.587	0.579

OR=odds-ratio; 95%CI=95% confidence interval; RACHS-1=risk adjustment for congenital heart surgery 1. Nagelkerke's R^2^ of 0.249. Mean standardized residuals of -0.004±0.956, *P*=0.921 from 0. Area under the receiver-operator characteristic (ROC) curve of 0.793, *P*<0.001 for the model.

A total of 508 surgeries could be attributed an ABC level. These were included in the model, which is shown in Table 5. In this model, the independent predictors of in-hospital death were ABC level 4 and age (≤ 30 days, and 30 days - 1 year).

**Table 5:** Multivariate logistic regression of in-hospital mortality using ABC levels (n=508).

	OR	95%CI		*P*-value
		Lower	Upper	
ABC level				0.139
1	1.000			-
2	1.956	0.733	5.219	0.181
3	2.138	0.786	5.819	0.137
4	3.811	1.219	11.913	0.021
Age group				0.000
≤ 30 days	8.658	3.936	19.044	0.000
30 days - 1 year	2.067	1.088	3.925	0.027
1 - 18 years	1.000			-
Prematurity	0.866	0.338	2.221	0.765
Major noncardiac structural anomaly	2.501	0.106	58.816	0.569
Combination procedure	2.051	0.843	4.993	0.113

OR=odds-ratio; 95%CI=95% confidence interval; ABC=Aristotle Basic Complexity Nagelkerke's R^2^ of 0.160. Mean standardized residuals of -0.007±0.966, *P*=0.859 from 0. Area under the receiver-operator characteristic (ROC) curve of 0.730, *P*<0.001 for the model.

Standardized mortality ratios were calculated for each of the two hospitals and are shown in Table 6.

**Table 6:** Standardized mortality ratios of the two participating centers (N=491).

Hospital	N	N'	Observed mortality	Predicted mortality	SMR
A	106	99	17.1%	14.6%	1.18
B	508	392	12.5%	13.1%	0.95
Total	614	491	13.4%		

N=total number of cases; N'=number of cases with an attributable RACHS-1 category; SMR=standardized mortality ratio

Outcomes Analysis: Infection

Overall incidence of infection was 13.8%, 26.8% being in hospital A (standardized infection ratio [SIR] of 1.58) and 10.9% in hospital B (SIR of 0.75). In a multivariate model, only RACHS-1 categories (3, 4, and 5/6) and age (≤ 30 days, and 30 days - 1 year) were independent predictors of any major infection (results not fully shown here).

## DISCUSSION

The costs of caring for a patient with CHD have been increasing year after year. Cost-effective strategies are therefore highly needed. However, this is not an easy task. Administrators, staff, families, and patients need to be in alignment, and the focus must be on transparency, performance, quality, safety, and commitment.

In fact, in 2014, pushed by the public and regulatory agencies, both the UK and the US expanded their public reporting of cardiac surgical outcomes^[10,11]^. There is hope that this initiative will result in more effective decision making in healthcare.

According to West^[12]^, there are three aims for achieving a sustainable, continuously improving healthcare system: better outcomes, better system performance, and better professional development. Sanchez & Barach^[13]^ stated that knowledge as well as cultural and organizational factors comprise the framework to thrive in cardiac care. Evolution in pediatric congenital cardiac care relies on the understanding of the relationships between the domains of outcomes analysis, quality improvement, and patient safety^[14]^. This can be achieved more easily with collaborative work such as ASSIST.

In fact, pediatric and congenital cardiac care and their associated outcomes have improved radically over the past generation in the developed world. This was accomplished, at least in part, by pursuing quality and performance, through careful analyses of local and collaborative databases of diagnoses, risk factors, procedures, complications, and outcomes, and searching for modifiable factors worsening the outcomes. This paper briefly described the process of implementing a data-driven quality improvement program in Brazil, along with challenges faced, solutions found, and preliminary data analysis of outcomes.

There are several similar approaches already in use by several countries: IQIC, PC4, STS-EACTS, among others. In fact, joining a collaborative database can result in several benefits. Jenkins et al.^[1]^ reported that, in 28 sites from 17 developing countries, joining IQIC resulted in a significant reduction in mortality rates. The impact of implementing a collaborative database focused on quality improvement was also reported in India by Balachandran et al.^[2]^. They reported that, after implementation of IQIC, there was a significant decrease in the incidence of bacterial sepsis and surgical site infection in addition to a significant decrease in intensive care unit length-of-stay. Mortality also decreased almost by half, but it did not reach statistical significance. Moreover, Sciarra et al.^[3]^ reported that implementation of initiatives such as IQIC is feasible in our region, improving the quality of care.

Quality Measures for Congenital and Pediatric Cardiac Surgery, developed and approved by The Society of Thoracic Surgeons (STS) and endorsed by the Congenital Heart Surgeons Society (CHSS), recommend participation in at least one multi-center, standardized data collection^[15]^. Organized according to Donabedian's Triad of Structure, Process, and Outcome, this initiative hoped that these quality measures can aid in congenital and pediatric cardiac surgical quality assessment and quality improvement initiatives.

In Brazil, the first efforts to create a quality improvement program aiming at outcomes of cardiac surgery began in 2014 for adult patients, encompassing the evaluation of institutional, team, and individual factors and focusing on the optimization of the existing resources^[16]^. However, at that time, there was no reliable source of information on outcomes after pediatric surgery for CHD. The only available source of information was the national database of all hospital admissions to public hospitals within the country: The National Health System database (DATASUS, http://datasus.saude.gov.br). This database was created with a focus on administrative, not clinical, points of view. This database is used to manage the public system, focusing on reimbursement to healthcare providers. It means that the information we have is based on what the providers inform they are doing, in terms of management, not epidemiological or clinical data.

We could have joined one of these successful approaches. In fact, many centers, individually, already have. However, after comprehensive discussions, our team decided to set up our own database. This decision was motivated by the feeling that we did not know enough about what we were doing, whether it was right or wrong. Joining an existing database would produce results in main outcomes and some basic demographics. Having a custom database would allow us to collect more data and, maybe, find out what worked best for each institution.

By highlighting the best practices for measuring outcomes, project ASSIST proposes a framework to help map out and support the next step in improving pediatric and congenital cardiac care in our country. This project started in the state of Sao Paulo and, in the future, it could become a national database. More than a database, the consortium will strengthen the relationship between centers around the country, building a new and better model of care and collaboration.

ASSIST has been developed philosophically and organizationally to follow the roadmap previously laid out by other successful, collaborative quality improvement pioneers, such as PC4, IQIC, among others. Transparency, introspection, and a commitment to rigorous science are the cornerstones of our approach, and the participating institutions are completely committed to these principles.

ASSIST was designed to facilitate the discovery of best practices among centers involved with CHD care and, using innovative research methods and collaborative learning, we expect to improve the overall results.

Nowadays, CHD represents the second cause of infant mortality in our state. This is our real challenge. ASSIST's mission is, therefore, to improve the number of treated patients and their outcomes, thus preventing potentially avoidable deaths.

Another issue that underwent a lot of discussion was the appropriateness of existing complexity scores to our reality. The options were the RACHS-1 score, the ABC score and level, and the STS-EACTS score. We did not know whether these scores would perform well in our patients. We found that, in our cohort, mortality was predicted by RACHS-1 with an AUC of 0.793, while ABC levels had an AUC of 0.730. In India, Joshi et al.^[17]^ evaluated the predictive value of RACHS-1 and ABC scores on mortality after surgery for CHD. They showed, in a cohort of 1150 patients, that the AUCs for mortality were: ABC score, 0.677; Aristotle Comprehensive Complexity (ACC) score, 0.704; and RACHS-1, 0.607. They concluded that ACC performed better over the other two. In our study, both RACHS-1 and ABC scores performed well. In Thailand, Vijarnsorn et al.^[18]^ reported the following AUCs for mortality in 230 patients: RACHS-1, 0.78; ABC, 0.74; and STS-EACTS, 0.67. These results were more similar to ours. In France, Bojan et al.^[19]^, in a cohort of 1384 patients, showed that AUCs for mortality were: RACHS-1, 0.75; and ACC, 0.87. In the largest dataset to date (45,635 patients), Jacobs et al.^[15]^ reported the following AUCs for mortality: ABC, 0.70; RACHS-1, 0.749; and STS-EACTS, 0.784.

In Brazil, Cavalcanti et al.^[20]^ investigated the accuracy of RACHS-1, STS-EACTS, and ABC scores in predicting mortality in a cohort of 360 patients who underwent surgery at a hospital in the state of Pernambuco, Brazil. This state is in the Northeastern part of Brazil, while ours is in the Southeastern. They showed that the three scores performed similarly, with the following AUCs: RACHS-1, 0.738; STS-EACTS, 0.739; and ABC, 0.766^[20]^. The results are very close to those obtained by us, even though the different regions of the country have very different socioeconomic statuses.

### Preliminary results and challenges they bring

The results of this preliminary analysis are of concern: we have a case mix very similar to others reported in the literature but, surprisingly, we found high mortality and infection rates, with significant differences between the first two institutions. Multivariate modeling showed that risk factors for death were higher RACHS-1 or ABC levels and age group (≤ 30 days, and 30 days - 1 year). The risk factors for major infection were the same. Those are non-modifiable factors. We hope that, by having a larger timespan of data, we will be able to identify other potentially modifiable factors contributing to poor outcomes.

### Limitations

This manuscript presents the very beginning of the idea of building our own CHD consortium, therefore, at this time there is no intention to deeply discuss the outcomes. The analysis of the collected data is used only to exemplify the potential of the ASSIST database. These results probably do not represent the actual performance of the centers, considering the short time frame of the data collected. Furthermore, perioperative care was not standardized. Variations in care are very likely to have happened. In addition, some cases were not included in the analysis because they were still hospitalized at the time of data harvest.

### Future

The next steps are:

(a) enroll more centers in the registry;
(b) refine the set of variables in order to keep only useful variables and thus save time and effort in collecting them; and
(c) start meetings to address main concerns, such as infection, and propose solutions.

The second phase of this project is to involve other centers in the state of Sao Paulo and, in a near future, others around the country, establishing a national database and collaborative network.

The purpose of ASSIST in promoting a multicenter database and collaboration will give us the opportunity to grow. The enormous progress that has occurred over the last several years to improve the quality and consistency of information about surgical treatment for congenital cardiac disease is not yet widespread in our country. Using a benchmarking assessment and a collaborative quality improvement approach, the centers involved with the pediatric cardiology care will build a strong partnership to achieve better outcomes. This initiative will define performance metrics that will encompass our gold standards of practice: diagnostics, management, technique, and follow-up.

## CONCLUSION

In conclusion, the ASSIST project was successfully created over a solid base of collaborative work. The main challenges faced and overcome were lack of institutional support, funding, computational infrastructure, dedicated staff, and trust. RACHS-1 and ABC scores performed well in our case mix. Our preliminary outcome analysis shows opportunities for improvement.

**Table:** 

Authors' roles & responsibilities	
FC	Substantial contributions to the conception or design of the work and grant proposal; final approval of the version to be published
PHM	Substantial contributions to the conception or design of the work and grant proposal; final approval of the version to be published
MNF	Data collection; final approval of the version to be published
NMI	Substantial contributions to the conception or design of the work and grant proposal; final approval of the version to be published
MBJ	Substantial contributions to the conception or design of the work and grant proposal; final approval of the version to be published
LA	Data collection; final approval of the version to be published
ALT	Substantial contributions to the conception or design of the work and grant proposal; data collection; final approval of the version to be published
LFC	Substantial contributions to the conception or design of the work and grant proposal; final approval of the version to be published
